# Science teachers’ integration of digital resources in education: A survey in rural areas of one Indonesian province

**DOI:** 10.1016/j.heliyon.2020.e04631

**Published:** 2020-08-05

**Authors:** Muhaimin, Asrial, Akhmad Habibi, Amirul Mukminin, Prosmala Hadisaputra

**Affiliations:** a Universitas Jambi, Indonesia; b LPDP Indonesia

**Keywords:** Education, Science teachers, PLS-SEM, Digital resources, Rural area

## Abstract

Digital resources can be implemented to support the quality of education, including for teachers teaching science. Studies have explored technology integration for schools in cities and urban areas; however, few studies focused on the technology integration in education of rural areas. Therefore, this study aimed at elaborating factors predicting Indonesian science teachers' integration of digital resources in education, especially for teaching activities in rural areas. Besides, demographic information was addressed to understand the differences regarding the integration of digital resources in education. We collected data from 217 respondents who are science teachers of senior and junior high schools in one province located in Sumatra, Indonesia. Using Smart PLS 3.0, we analyzed the data to understand the relationship between exogenous and endogenous variables. In addition, t-test was used to elaborate on the differences regarding the integration of digital resources based on demographic information. Findings from the path analysis show that attitude was the strongest driver predicting intention to use digital resources in education perceived by teachers in rural areas. Meanwhile, self-efficacy was reported to be insignificant for the intention to use. The only predictor that is positively significant for actual behavior was intention to use. Other factors (facilitating condition and knowledge and skills) did not affect actual behavior. In addition, the difference test informed that Mean values between participants’ teaching experience was significantly different regarding the integration of digital resources of schools in rural areas. No significant differences were reported based on gender and level of school. The technology integration for schools in rural areas is different compared to the schools in cities and urban areas. With limited accesses and resources, this study might provide practical implications and recommendations for further research, especially on technology integration in schools of rural area.

## 1 Introduction

Nowadays, experts in education have almost similar academic opinions that people around the world should have free, open, and easy access to reach digital resources for the purpose of teaching and learning (Hoosen, 2012). Recently, many countries make maximal efforts in the investment to improve the establishment of digital resources for education, especially for teachers. The technology potentiality should be more developed. Studies have informed that the digital resources for education have been well-implemented in developed countries such as USA (Tyler-Wood et al., 2018), Norway (Instefjord and Munthe, 2017), and China (Wang et al., 2019).

This potential has also been made to be suitable with major cities in developing countries, for example, Jakarta as the capital city of Indonesia and Kualalumpur in Malaysia. However, the questions emerged whether similar conditions arise for teachers working in schools of rural areas. In terms of educational quality, teachers in rural areas seem to be less qualified than that of their urban peers, teachers working in major cities (Liu and Onwuegbuzie, 2012). In addition, the lack of quality resources and their access to digital technology have been recognized as pressing challenges (Habibi et al., 2020).

Regarding to theses premises, one of the countries that still has many challenges in implementing digital resources in rural areas’ education is Indonesia. As a country with more than 260 millions people, technology has reached almost 60 % of the total population and made it essential to maximize the use of technology in education. Even though the gap of social and economic exist between urban and rural areas, the equal opportunity should be handed to all students including in terms of access to technology-based education (Qian and Smyth, 2008). Commonly, the factors that are linked with the differences in the quality of education is digital resources for teaching, considered as one of the key factors. The educational policy that tends to prioritize big cities over rural areas should be the main concern (Shah, 2016) since it could result in a big gap.

Many studies have reported teachers' educational technology integration in major cities (Farjon et al., 2019; Habibi et al., 2020). However, few researchers elaborated technology integration perceived by teachers from rural areas (Tyler-Wood et al., 2018; Qian and Smyth, 2008). Even fewer studies reported the integration in developing countries like Indonesia. Therefore, this study is proposed to elaborate on factors predicting Indonesian teachers' integration of digital resources in rural areas into their teaching activities. Demographic information was also addressed to understand the difference regarding the integration. Specifically, we addressed this study to science teachers for a common in-depth and specific understanding of the phenomenon. The findings of this study might have a useful contribution to teachers’ professional development plan and initiatives regarding the integration of digital resources in education.

## 2 Theoretical framework

**Figure 1:**
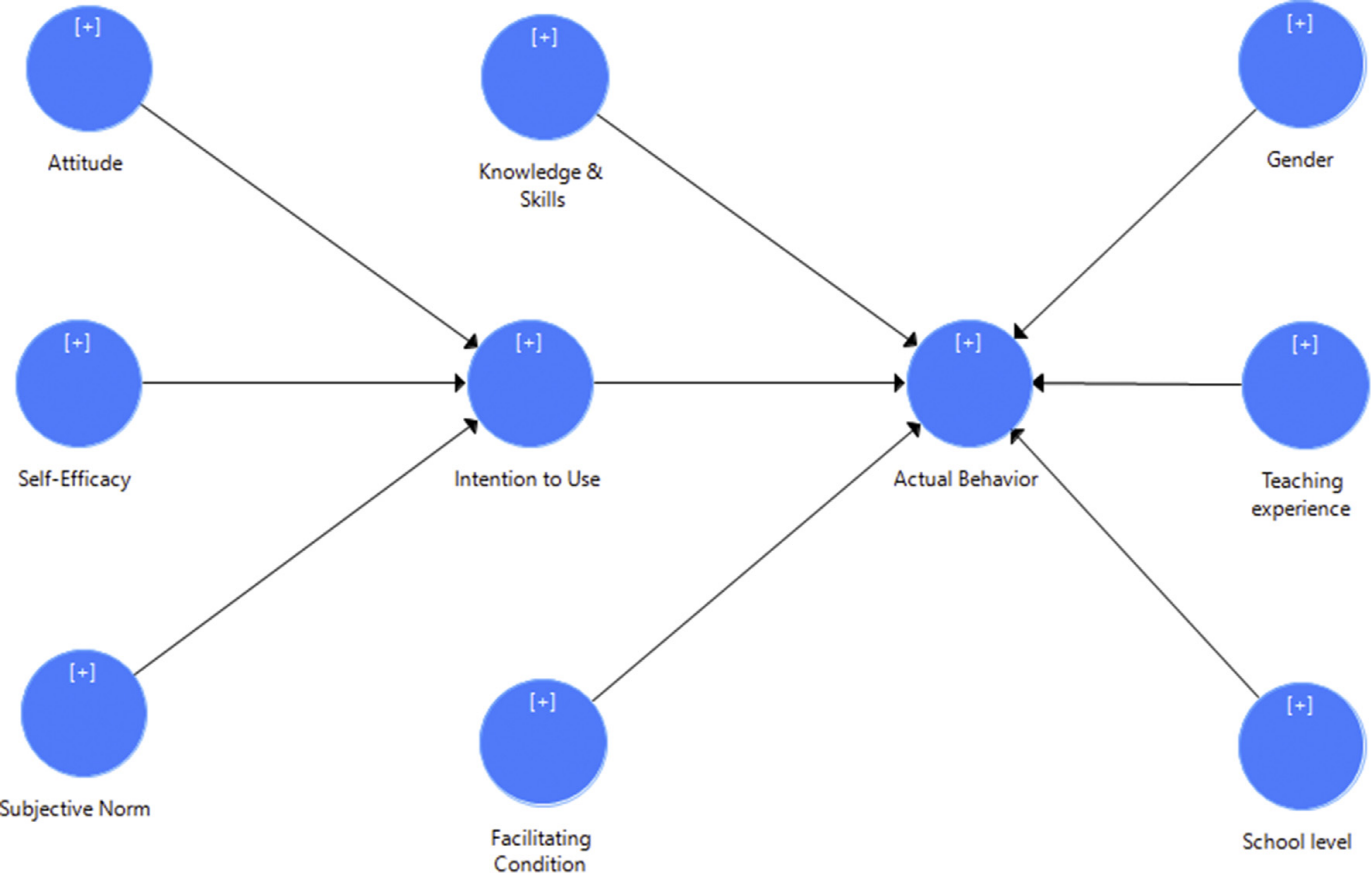
Proposed model.

Some frameworks have been established that focused on technology integration in education, such as Technological Pedagogical Content Framework, TPACK (Koehler and Mishra, 2009); Technology Acceptance Model, TAM (Davis et al., 1989); Technology Integration Assessment Instrument, TIAI (Britten and Cassady, 2005); Unified Theory of Technology Acceptance Model, UTAUT (Venkatesh et al., 2003). However, to obtain an in-depth analysis of factors predicting Indonesian science teachers' integration of digital resources in school of rural areas, the Fishbein and Ajzen (2011) Integrative Model of Behavior Prediction (IMBP) framework was used. The selection of IMBP as the proposed model of this study was discussed among researchers and with three Indonesian educational technology experts. The framework includes three key factors (attitude, self-efficacy, and subjective norm) predicting intention to use. In addition, knowledge and skills, intention to use, and facilitating condition were hypothesized to have a significant relationship with actual behavior. Previous studies informed that IMBP is a valid and reliable model to explain teachers' integration of different types of technology in education (Vermeulen et al., 2017; Wang et al., 2019). To support the framework, demographic information (gender, teaching experience and school level) was included for the difference analysis regarding the actual behavior (see Figure 1).

### 2.1 Attitude

Attitude in this study is defined as Indonesian science teachers’ general feeling of sympathy or antipathy toward the integration of digital resources in education. Many studies have informed a correlation between teachers' attitudes and technology integration in pedagogy. They mostly reported that the more teachers believe that the integration of technology is a good activity, the more they want to integrate it in their instructional activities, such as Admiraal et al. (2017) for hardware facilities, Kreijns et al. (2013) for digital learning materials, and Muhaimin et al. (2019) for Web 2.0. However, Aslan and Zhu (2017) reported a non-significant relationships between attitude and technology integration in instructional activities. Similarly, Estriegana et al. (2019) informed attitude toward technology integration was not strongly related to virtual laboratory acceptance and practical work.

### 2.2 Self-efficacy

Self-efficacy in this study refers to the Indonesian science teachers’ belief in their own capabilities to perform teaching by integrating digital resources in education. Theoretically, when teachers have confidence to integrate technology into their teaching activities, they will more likely use it on a daily basis (Mlambo et al., 2020). Earlier studies have reported the strong relationship between self-efficacy and intention to use or actual behavior to use technology into teaching (Lee and Lee, 2014; Rohatgi et al., 2016). For instance, a finding from Van Acker, Vermeulen, Kreijns, Lutgerink, and Van Buuren (2014) reported that self-efficacy significantly predicted actual behavior of the use of technology. On the other hand, Teo et al. (2018) through their research informed insignificant relationship between self-efficacy and intention to use Web 2.0.

### 2.3 Subjective norm

In this study, subjective norm is perceived pressures felt by Indonesian science teachers from their important people to integrate digital resources in their teaching activities. In the previous studies, the positive correlation was reported between subjective norms and technology use (Muhaimin et al., 2019; Yau and Ho, 2015). For example, Yau and Ho (2015) informed that subjective norm significantly predicted the intention to use in using e-learning. However, no significant relationship between subjective norm and intention to use digital learning management (Kreijns et al., 2017). In a meta analysis study conducted by Schepers and Wetzels (2007), subjective norm was not a key driving factor affecting behavioral intention and use of technology in four included studies.

### 2.4 Knowledge and skills

IMBP was extended by postulating knowledge and skills to predict actual behavioral (Fishbein and Ajzen, 2011). In this study, knowledge and skills are hypothesized to be an antecedent of actual behavior. In this context, if teachers have the competence to integrate digital resources in their teaching activities, they will likely improve the integration. Wang et al. (2019) found that knowledge and skill has a significant positive relationship with digital educational resources. Habibi et al. (2020) also found a similar finding, a strong relationship between knowledge and the use of ICT among pre-service teachers.

### 2.5 Intention to use

Intention to use is an indication of an individual's readiness to perform a given behavior (Ajzen, 1991). In this study, it is hypothesized to significantly predict actual behavior regarding the integration of digital resources in education. The studies about the intention to use and actual behavior are still limited. One of the studies informed that no correlation emerged between behavior intention and actual behavior regarding digital educational resources (Wang et al., 2019). Therefore, we also included this relationship for the 5^th^ hypothesis.

### 2.6 Facilitating conditions

Facilitating conditions in this study are defined as the degree to which Indonesian science teachers believe that organizational and technical resources exist to support the integration of digital resources in education in Indonesian rural areas. In this context, facilitating condition may relate to technological infrastructure access, professional development availability, technical support and lead, and supporting policies promoting the integration of technology into teaching. Teo (2009) reported a positive link between facilitating condition and intention to use technology among pre-service teachers’ intention to use technology in their teaching practices. Wang et al. (2019) reported the existence of a correlation between facilitating condition and use of digital resources.

### 2.7 Demographic information

In addition to factors affecting the integration of digital resources in education, three types of demographic information, namely gender, teaching experience, and school level, were included to understand the difference regarding the actual integration. Many studies have reported to understand the relationship between domographic information and technology integration (e.g. Aslan and Zhu, 2017; Ramírez-Correa et al., 2014; Whitley, 1997; Yuen and Ma, 2002). Gender was reported to be significantly different regarding multimedia technology adoption for learning (Ramírez-Correa et al., 2014) and computer acceptance (Yuen and Ma, 2002; Whitley, 1997). Teaching objects were also reported to be different in their relationship with the use of ICT among Turkish pre-service teachers (Aslan and Zhu, 2017).

## 3 Methods

The current study follows a survey research design, a quantitative approach. A Survey design is a procedure in quantitative culture providing the opportunity for researchers to administer questionnaires to sample or entire population in order to elaborate their attitudes, perception, and behavior (Creswell, 2014). Some steps were done to analyze the data, namely content validity, measurement model, and structural model as well as t-test. Informed consent was achieved from all participants who have been involved in the study and the researchers ensured their anonymity. Universitas Jambi and LPDP Indonesia that provide the funding for this research did not require any ethical approval for this study. Nine hypotheses were formulated;

H1 Indonesian science teachers' attitude toward the integration of digital resources in education will have a significant effect to Intention to Use.

H2 Indonesian science teachers' self-efficacy toward the integration of digital resources in education will have a significant effect on intention to use.

H3 Indonesian science teachers' subjective norm toward the integration of digital resources in education will have a significant effect on intention to use.

H4 Indonesian science teachers' knowledge and skills on the integration of digital resources in education will have a significant effect on actual behavior.

H5 Indonesian science teachers' intention to use toward the integration of digital resources in education will have a significant effect on actual behavior.

H6 Indonesian science teachers' perceived of facilitating condition the integration of digital resources in education will have a significant effect on actual behavior.

H7 There will be a significant difference of the actual behavior of Indonesian science teachers' integration of digital resources in education based on gender.

H8 There will be a significant difference of the actual behavior of Indonesian science teachers' integration of digital resources in education based on teaching experience.

H9 There will be a significant difference of the actual behavior of Indonesian science teachers' integration of digital resources in education based on levels of school.

### 3.1 Research context

The study aimed to elaborate on factors predicting Indonesian science teachers’ integration of digital resources in education in rural areas and to understand the differences regarding the integration of digital resources based on three demographic information. Rural area is an area with a low population density and small settlements, commonly agricultural areas are commonly rural. Based on these criteria, forty per cent of Indonesian people live in rural areas (IFAD, 2017). In the current study, two rural areas located in central Part of Sumatra Island, Indonesia were chosen. Most people in these areas work as palm plantation farmers. Within these two areas, there are 165 schools with 1731 teachers who are the target population of this study. Research accessibility and financial supporting are the two main reasons of the selection of the target population (Van Wee, 2016).

### 3.2 Instrumentation

The review of literature support researchers with definitions and analyses of the theories and concepts for theoretical framework elaboration (Prasojo et al., 2020). It also helps researchers choose methods and instruments (Hair et al., 2016). The survey instrument of this study was adapted from previous studies (Table 1). There were thirty items generated in this stage. To suit the context and setting of the study, face and content validity with five users and five experts were conducted. In this process, two items of attitude and one item of knowledge and skills were dropped. Afterwards, the instrument was piloted to 45 teachers aiming to evaluate the reliability. Through Cronbach's alpha test (Table 1), the instrument was reliable for the main data collection. To ease the whole process of the instrumentation, data collection and data analysis, we used back translation (Behr, 2017). The instrument was translated from English to Indonesian language and vice versa involving two Indonesian doctoral students who have attended English translation courses. All items were measured using a 5-point Likert Scale (1 = very disagree, 2 = disagree, 3 = neutral, 4 = agree to 5 = very agree). Besides, demographic questions were also included. From the pilot study data analysis process, 27 indicators remained for the main data collection (Table 1).

**Table 1:** Items generated after the reliability test in the Pilot study.

Construct	Items	Sample	Adapted from	α
Attitude	AT1, AT2, AT3	AT1. Using digital resources is a good idea for teaching science.	(Admiraal et al., 2017; Wang et al., 2019)	0.712
Self-efficacy	SE1, SE2	SE2. If students have questions about digital resources, I can help them.	(Wang et al., 2019)	0.817
Subjective norms	SN1, SN2, SN3, SN4	SN3. My school principal supports the use of digital resources.	(Muhaimin et al.,. 2019; Teo et al., 2018)	0.823
Knowledge and skills	KS1, KS2, KS3, KS4	KS4. I can use digital resources in a variety of teaching activities.	(Habibi et al., 2020; Wang et al., 2019)	0.751
Intention to use	IT1, IT2, IT3	IT1. I will use digital resources in teaching science.	(Wang et al., 2019)	0.872
Facilitating condition	FC1, FC2, FC3, FC4	FC2. Guidance is available for me in selecting digital resources for teaching.	(Muhaimin et al.,. 2019; Teo et al., 2018)	0.767
Actual behavior	AB1, AB2, AB3, AB4, AB5, AB6, AB7	AB3. How often do you use multimedia material (text, image, animation, video, audio, etc.) in teaching science.	(Wang et al., 2019)	0.851

### 3.3 Data collection and preparation

We contacted the head of the educational department of the two rural areas to have the access for the data collection. Using stratified random sampling, we sent official letters to all 50 schools representing the location and school setting. We distributed printed instrument for science teachers who taught Chemistry, Biology, and Physics. Prior the distribution, we explained the definition and examples of digital resources in education. Demographic information of all participants of the current study is informed in Table 2.

**Table 2:** Demographic information.

Demography (n.217)	Sub-category	Frequency	%
Gender	Female	127	58.5
	Male	90	41.5
Teaching experience	Less than 5 years	64	29.5
	More than five years	153	70.5
School level	Junior high school	149	68.7
	Senior high school	68	31.3

### 3.4 Data analysis

In the main data analysis, we applied Partial Least Square- Structural Equation Modeling (PLS-SEM) with Smartpls 3 software since it was described to have more solutions with small sample sizes of model with many constructs and to test (Willaby et al., 2015). In addition, PLS-SEM is convenient when the goal of the research is to examine the extension of an existing theory (Hair et al., 2016). Two steps of PLS-SEM were included, reflective measurement model assessment and structural model assessment (Gao and Huang, 2019; Hair et al., 2019). The reflective measurement model assessment was reported through reflective indicator loading, internal consistency reliability consisting of Cronbach's alpha and Composite Reliability, convergent validity through Average Variance Extracted, and discriminant validity using Heterotrait-Monotrait ratio or HTMT. The structural model assessment was measured using some statistical assessments; VIF value, path coefficients, t statistics, and *p* value. T-test was used to understand the difference regarding the actual behavior to use digital resources among Indonesian science teachers in rural areas based on gender, teaching experience, and school level.

## 4 Findings

### 4.1 Reflective measurement model

For the reflective measurement model assessment, we reported reflective indicator loading (>0.708), internal consistency reliability consisting of Cronbach's alpha and Composite Reliability (0.700–0.900), convergent validity through Average Variance Extracted (>0.500), and discriminant validity using HTMT (>0.900). These thresholds were adopted from Hair et al. (2019). From the assessment of the reflective measurement model, some loading values were informed to be less than the threshold values of 0.700. All values with reflective loading values of 0.708 were subsequently deleted from the model (Hair et al., 2019). There were six items dropped from the process (IT2, AB3, AB4, AB5, AB6, AB7). All information regarding the final assessment of lading, Cronbach's alpha, Composite Reliability, Average Variance Extracted, and HTMT can be seen in Tables 3 and 4. The model is valid and reliable for the assessment of the structural model.

**Table 3:** Summary results for the reflective constructs of the measurement model.

Construct	Items	Loading	Cronbach's Alpha	Composite Reliability	Average Variance Extracted
Actual behavior	AB1	0.909	0.703	0.869	0.768
	AB2	0.843			
Attitude	AT1	0.860	0.864	0.917	0.786
	AT2	0.907			
	AT3	0.892			
Facilitating condition	FC1	0.801	0.865	0.907	0.709
	FC2	0.786			
	FC3	0.884			
	FC4	0.892			
Intention to use	IT1	0.875	0.706	0.872	0.773
	IT3	0.883			
Knowledge & skills	KS1	0.883	0.872	0.912	0.721
	KS2	0.897			
	KS3	0.783			
	KS4	0.829			
Self-efficacy	SE1	0.920	0.735	0.881	0.788
	SE2	0.854			
Subjective norm	SN1	0.844	0.870	0.911	0.720
	SN2	0.881			
	SN3	0.807			
	SN4	0.860

**Table 4:** HTMT.

	Actual behavior	Attitude	Facilitating Condition	Intention to Use	Knowledge & Skills	Self-Efficacy
Attitude	0.324					
Facilitating condition	0.382	0.609				
Intention to use	0.560	0.592	0.888			
Knowledge & skills	0.306	0.419	0.825	0.789		
Self-efficacy	0.328	0.612	0.640	0.516	0.475	
Subjective norm	0.359	0.504	0.766	0.815	0.852	0.543

### 4.2 Structural model assessment

We used a bootstrapping procedure with 5,000 sub-samples with 217 cases. Two-tailed *t*-test to evaluate the significance of the coefficients was then addressed. However, Collinearity of each predicting relationship should be below 3 for the inner Variance Inflation Factor (VIF). The VIF value was processed through the computation of multiple regression as recommended by Hair et al. (2019). All relationship values of VIF meet the requirement (Table 5).

**Table 5:** VIF value.

Facilitating Condition	Intention to Use
Attitude → Intention to use	1.444
Facilitating condition → Actual behavior	2.490
Intention to use→ Actual behavior	2.038
Knowledge & skills → Actual behavior	2.101
Self-efficacy → Intention to use	1.448
Subjective norm → Intention to use	1.344

**Figure 2:**
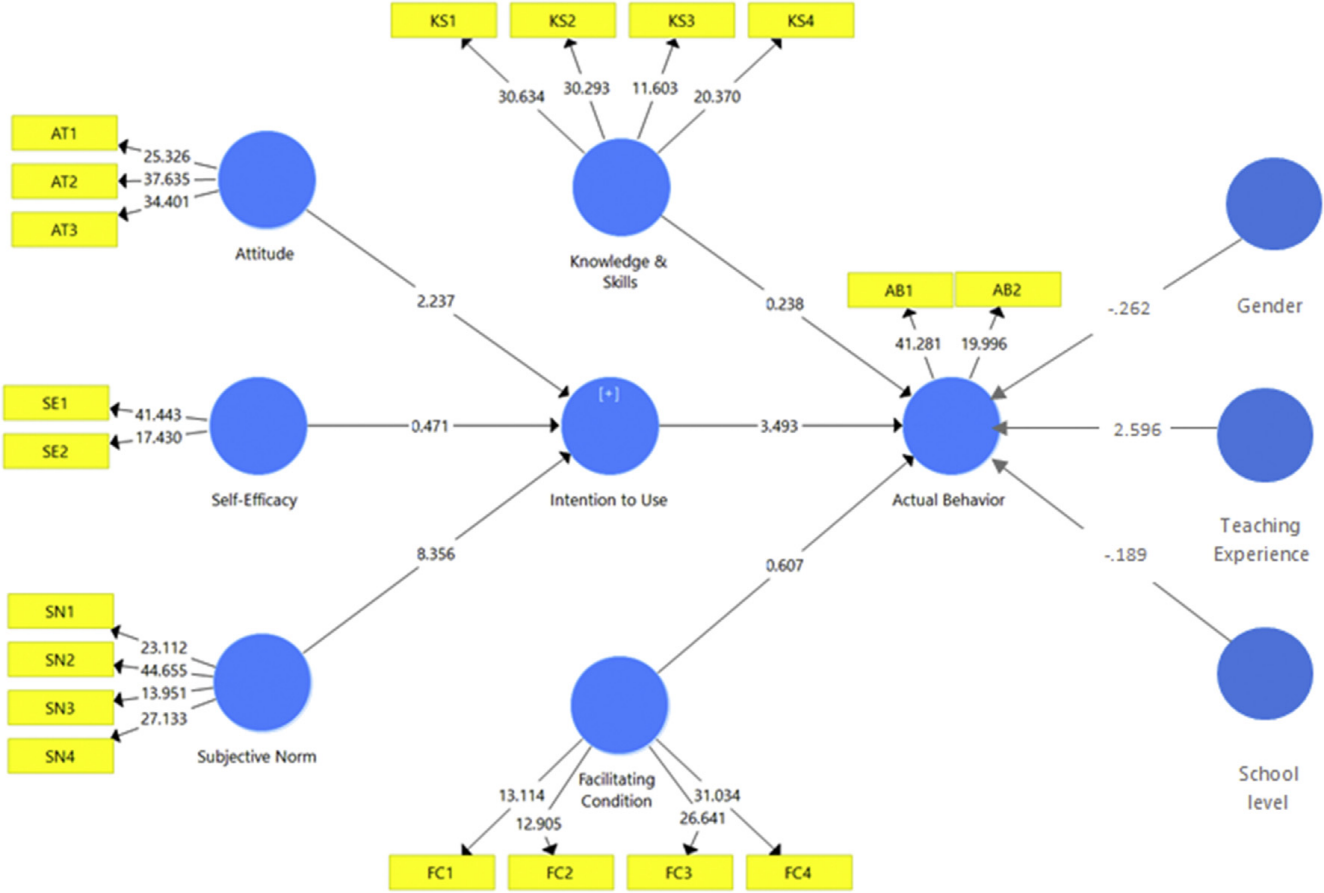
Final model.

Since the VIF values have met the threshold, we processed the data to investigate the path coefficient. Setting the two-tailed test at the level of .05, the report of this study informed that three relationships are significant; the three others are not significant. In predicting intention to use, attitude is a significant predictor (*β* = 0.213; t statistics = 2.237). Subjective norm is the most significant key predictor for intention to use (*β* = 0.531; t statistics = 8.356). In addition, the most significant factor for the actual behavior regarding the integration of digital resources among Indonesian science teachers is intention to use (*β* = 0.360; t statistics = 3.439). Table 6 informs the complete results of the bootstrapping process. Meanwhile, Figure 2 shows the t statistics and the loading of each indicator.

**Table 6:** Bootstrapping results.

Path	β	Sample mean (M)	t statistics	*p* values	Significance
Attitude -> Intention to use	0.213	0.234	2.237	*p* < .05	Yes
Facilitating condition -> Actual behavior	0.086	0.085	0.607	0.544	No
Intention to use -> Actual behavior	0.360	0.357	3.493	*p* < .05	Yes
Knowledge & skills -> Actual behavior	-0.030	-0.017	0.238	0.812	No
Self-efficacy -> Intention to use	0.038	0.029	0.471	0.637	No
Subjective norm -> Intention to use	0.531	0.530	8.356	*p* < .05	Yes

### 4.3 Differences regarding the integration of digital resources in education based on demographic information

In addition to the structural model reported in this study, we investigated whether the demographic information (gender, teaching experience, and school level) are different regarding the actual behavior of the integration of digital resources in education. Using t-test, the results informed that no significant differences emerged between gender and level of school. However, teaching experience is informed to be significantly different regarding the actual behavior (t = 2.596; *p* < 0.05). Complete details of the t-test result are informed in Table 7 and Figure 2.

**Table 7:** T-test result.

Demographic		n	Mean	Mean Difference	F	Sig.	t	Df	*p* value
Gender	Female	127	3.972	-.01645	.810	.369	-.262	215	.794
	Male	90	3.989						
Experience	>5 years	64	4.102	.17346	.772	.380	2.596	215	.010
	<5 years	153	3.928						
School level	Junior high	149	3.971	-.01263	9.741	.002	-.189	215	.850
	Senior high	68	3.983						

## 5 Discussion

In order to support quality education, digital resources can be used and applied to teaching and learning activities (Habibi et al., 2020). Urban and rural areas should differed regarding the policy on the development of digital resources in education. In this study, we adapted instruments from previous studies (Admiraal et al., 2017; Habibi et al., 2020; Muhaimin et al., 2019; Teo et al., 2018; Wang et al., 2019). In validating the instrument, we attributed content validity where three items were removed since they were not suitable for the Indonesian context. Meanwhile, through a pilot study, we evaluated the reliability of the study. After the processes, seven constructs with twenty-seven items were included in the main data collection. After the main data collection, we assessed the reflective measurement model through the procedures suggested by Hair et al. (2019). Some measurements, such as reflective indicator loadings, internal consistency reliability, convergent validity, and discriminant validity were measured. In this process, five items were eliminated resulting twenty-one items for the assessment of the structural model by measuring the path coefficient, t statistics, and *p* values.

From the assessment of the structural model, three relationships were reported to be significant. Regarding the intention to use digital resources in education, subjective norm was the strongest predictor. Subjective norm has been reported to significantly predict intention to use or actual use of technology in education (Muhaimin et al., 2019; Yau and Ho, 2015; Wang et al., 2019). This is proof that the people around the participants of this study had influenced them in determining their intention to use digital resources in education. The finding might also be triggered by the culture of the Indonesian who put highly on respecting of other people opinions and acts. This culture has been historically used by people not only from the province where this study was conducted but also other provinces in all six major Islands in Indonesia (Sumatra, Java, Bali, Sulawesi, Kalimantan, and Papua).

In addition to subjective norm, attitude also has a significant role to be one of the key drivers predicting intention to use digital resources in education. In the Indonesian context, attitude has been reported to predict the integration of technology in education; Muhaimin et al. (2019) informed that it influence Web 2.0 integration among Indonesian pre-service teachers. Similarly, other studies have also informed similar results (Admiraal et al., 2017; Kreijns et al., 2013). This finding refers to the positive feeling of the participants in improving the digital resources integration in education among Indonesian science teachers. In this study, the only factor that is not significant in predicting intention to use digital resources in education is self-efficacy. This finding is incompatible with the results by Van Acker et al. (2014) who underlined a strong link between self-efficacy and actual behavior. However, the finding strengthens Teo’s et al (2018) who informed a non significant relationship between self-efficacy and intention to use Web 2.0 among Chinese pre-service teachers. In brief, Indonesian science teachers beliefs on their abilities to do teaching with digital resources have no link with the integration.

For actual behavior regarding the integration of digital resources in education among Indonesian science teachers in rural areas, intention to use is the only predictor reported to be significant. The intention to use and actual use of technology has been continuously reported to be related. By having a strong intention to use digital resources in education, Indonesian teachers’ use of this technology improves. Meanwhile, the other two hypostasized factors (facilitating condition; knowledge and skills) do not significantly predict the actual use of digital resources perceived by the participants. These results are not in line with what other studies revealed. For example, Habibi et al. (2020) found that knowledge strongly affected the use of information and communication technology during teaching practice. In addition, Teo (2009) found similar result between facilitating condition and actual use of technology. These two findings can be described as the lack of training and infrastructures for improving teachers use of technology in rural areas.

Besides factors affecting the integration of digital resources in education, the current study also investigated the role of age, teaching experience, and school level. Based on the t-test, experience between new teacher and senior teachers is different. New teachers tend to use digital resources more frequently than those of their older counterparts. This finding is evidence that old teachers of Indonesian teachers teaching science might find it difficult to use technology in their teaching. The familiarity with various kinds of technology might also be one of the factors which make Indonesian new teachers are competitive using digital resource. This difference is in contrast with previous studies reporting senior teachers were more frequent in using technology (Van Acker et al., 2014). Surprisingly, no significant differences were found based on gender and school level which show that is different with other studies (Aslan & Zhu, 2017; Ramírez-Correa et al., 2014). Further studies should be carried out regarding demographic information differences for technology integration in rural areas’ schools since similar phenomenon has been reported by many researchers for urban schools.

## 6 Conclusion

As a country with more than 17,000 Islands, common weaknesses of the spread rural areas in Indonesian include weak quality of human resources, uneven physical infrastructure, ownership of capital, insufficient provision of social safety, low effectiveness and efficiency of local government spending, bias policies from central government, remoteness, and conflicts could be overcome with appropriate policies and financial spending (Resosudarmo, 2015). Similar characteristics of Indonesian rural areas across the Islands e.g. Sumatra, Java, Bali, Sulawesi, Kalimantan, and Papua could make a greater contribution of the findings for the betterment of Indonesian education, especially in rural areas. The contribution of the current study is especially important for the perspective of technology integration in rural areas for science teachers.

From the findings of the study, the role of social pressures is informed as a key predicting factor for the intention to use digital resources perceived by teachers in rural areas. Therefore, the facilitation of teachers' group for mutual encouragement for technology integration is required. In addition, attitude toward the integration of digital resources in education significantly predict the intention to use. This positive relationship shows that science teachers in rural areas believe that the use of technology in education is important. The implication can be a trigger for further steps in improving technology integration in rural schools; teachers beliefs toward technology integration can be insightful materials for every step taken for educational technology implementation for both local and national schools of every level. Therefore, science teachers’ attitude in rural schools should always be supported regarding the use of digital resources. Lastly, intention to use digital resource among science teachers in rural areas is the only predictor reported to be significant for actual behavior. Thus, improving the intention will likely affect the real use of technology among the science teachers.

Considering the findings of the current study, related stake holders might conduct professional development plan and initiatives to improve teachers' experience, knowledge, and information for the integration of digital resources in education. Trainings and seminars should be improved, especially for senior rural area teachers in teaching science who are not accustomed to using technology in teaching. To promote this, technological pedagogical and content knowledge for science teachers could be implemented as well as for other subject matter; to effectively integrate technology in education, it requires sensitivity establishment to the relationship between technology, pedagogy and content. Teachers as individuals, school level, teaching experience, context, and cultures as well as other factors, should also be a unique consideration for the improvement. In general, the findings of this study can be a guideline for stakeholders of rural areas schools, especially in developing countries, to set appropriate policies for the betterment of technology integration. School principals should keep motivating their teachers’ use of technology during teaching and teachers should also mutually encourage their peers. The findings also indicate that supporting facilities and teachers knowledge is still limited; therefore, funding needs to be improved. Corporate social responsibility from private companies can also support the integration. Further studies within other contexts and setting are recommended to conduct. Besides, other methods of data collection, such as observation and interview are also suggested.

## Declarations

### Author contribution statement

M. Muhaimin: Conceived and designed the experiments; Performed the experiments; Wrote the paper.

A. Asrial: Conceived and designed the experiments; Contributed reagents, materials, analysis tools or data; Wrote the paper.

A. Habibi: Conceived and designed the experiments; Contributed reagents, materials, analysis tools or data; Analyzed and interpreted the data; Wrote the paper.

A. Mukminin: Conceived and designed the experiments; Contributed reagents, materials, analysis tools or data.

P. Hadisaputra: Contributed reagents, materials, analysis tools or data; Wrote the paper.

### Funding statement

This work was supported by Universitas Jambi and 10.13039/501100014538Lembaga Pengelola Dana Pendidikan (LPDP) Indonesia.

### Competing interest statement

The authors declare no conflict of interest.

### Additional information

No additional information is available for this paper.

## References

[bib1] Admiraal W., Louws M., Lockhorst D., Paas T., Buynsters M., Cviko A., Jansen C., Jonge M., Nouwens S., Post L., van der Ven F., Kester L. (2017). Teachers in school-based technology innovations: a typology of their beliefs on teaching and technology. Comput. Educ..

[bib2] Ajzen I. (1991). The theory of planned behavior. Organ. Behav. Hum. Decis. Process..

[bib3] Aslan A., Zhu C. (2017). Investigating variables predicting Turkish pre-service teachers' integration of ICT into teaching practices. Br. J. Educ. Technol..

[bib4] Behr D. (2017). Assessing the use of back translation: the shortcomings of back translation as a quality testing method. Int. J. Soc. Res. Methodol..

[bib5] Britten J.S., Cassady J.C. (2005). The Technology Integration Assessment Instrument: understanding planned use of technology by classroom teachers. Comput. Sch..

[bib6] Creswell J.W. (2014). Research Design: Qualitative, Quantitative, and Mixed Methods Approaches.

[bib7] Davis F.D., Bagozzi R.P., Warshaw P.R. (1989). User acceptance of computer technology: a comparison of two theoretical models. Manag. Sci..

[bib8] Estriegana R., Medina-Merodio J.A., Barchino R. (2019). Student acceptance of virtual laboratory and practical work: an extension of the technology acceptance model. Comput. Educ..

[bib9] Farjon D., Smits A., Voogt J. (2019). Technology integration of pre-service teachers explained by attitudes and beliefs, competency, access, and experience. Comput. Educ..

[bib10] Fishbein M., Ajzen I. (2011). Predicting and Changing Behavior: the Reasoned Action Approach.

[bib11] Gao B., Huang L. (2019). Understanding interactive user behavior in smart media content service: an integration of tam and smart service belief factors. Heliyon.

[bib12] Habibi A., Yusop F.D., Razak R.A. (2020). The role of TPACK in affecting pre-service language teachers’ ICT integration during teaching practices: Indonesian context. Educ. Inf. Technol..

[bib13] Hair J.F., Hult G.T.M., Ringle C., Sarstedt M. (2016). A Primer on Partial Least Squaress Structural Equation Modeling (PLS-SEM).

[bib14] Hair J.F., Risher J.J., Sarstedt M., Ringle C.M. (2019). When to use and how to report the results of PLS-SEM. Eur. Bus. Rev..

[bib15] Hoosen S. (2012). *Survey on Governments' Open Educational Resources (OER) Policies* Vancouver/Paris.

[bib16] IFAD (2017). Investing in Rural Areas Investing in Indonesia.

[bib17] Instefjord E.J., Munthe E. (2017). Educating digitally competent teachers: a study of integration of professional digital competence in teacher education. Teach. Teach. Educ..

[bib18] Kreijns K., Van Acker F., Vermeulen M., Van Buuren H. (2013). What stimulates teachers to integrate ICT in their pedagogical practices? The use of digital learning materials in education. Comput. Hum. Behav..

[bib19] Kreijns K., Vermeulen M., Buuren H.V., Acker F.V. (2017). Does successful use of digital learning materials predict teachers’ intention to use them again in the future?. Int. Rev. Res. Open Dist. Learn..

[bib20] Koehler M., Mishra P. (2009). What is technological pedagogical content knowledge (TPACK)?. Contemp. Issues Technol. Teach. Educ..

[bib21] Lee Y., Lee J. (2014). Enhancing pre-service teachers' self-efficacy beliefs for technology integration through lesson planning practice. Comput. Educ..

[bib22] Liu S., Onwuegbuzie A.J. (2012). Chinese teachers' work stress and their turnover intention. Int. J. Educ. Res..

[bib23] Mlambo S., Rambe P., Schlebusch L. (2020). Effects of Gauteng province’s educators’ ICT self-efficacy on their pedagogical use of ICTS in classrooms. Heliyon.

[bib24] Muhaimin Habibi A., Mukminin A., Pratama R., Asrial, Harja H. (2019). Predicting factors affecting intention to use web 2.0 in learning: evidence from science education. J. Baltic Sci. Educ..

[bib25] Prasojo L.D., Habibi A., Yaakob M.F.M., Pratama R., Yusof M.R., Mukminin A., Hanum F. (2020). Teachers’ burnout: a SEM analysis in an Asian context. Heliyon.

[bib26] Qian X., Smyth R. (2008). Measuring regional inequality of education in China: widening coast–inland gap or widening rural–urban gap?. *J. Int. Dev.*: J. Dev. Sustain. Agric..

[bib27] Ramírez-Correa P., Rondán-Cataluña F.J., Arenas-Gaitán J. (2014). An empirical analysis of mobile Internet acceptance in Chile. Inf. Res..

[bib28] Resosudarmo B.P. (2015). Rural Development in Indonesia.

[bib29] Rohatgi A., Scherer R., Hatlevik O.E. (2016). The role of ICT self-efficacy for students' ICT use and their achievement in a computer and information literacy test. Comput. Educ..

[bib30] Schepers J., Wetzels M. (2007). A meta-analysis of the technology acceptance model: investigating subjective norm and moderation effects. Inf. Manag..

[bib31] Shah A. (2016). Fiscal policies for coordinated urban-rural development and their relevance for China. Publ. Finance Manag..

[bib32] Teo T. (2009). Modelling technology acceptance in education: a study of pre-service teachers. Comput. Educ..

[bib33] Teo T., Sang G., Mei B., Hoi C.K.W. (2018). Investigating pre-service teachers’ acceptance of Web 2.0 technologies in their future teaching: a Chinese perspective. Interact. Learn. Environ..

[bib34] Tyler-Wood T.L., Cockerham D., Johnson K.R. (2018). Implementing new technologies in a middle school curriculum: a rural perspective. Smart Learn. Environ..

[bib35] Van Acker F., Vermeulen M., Kreijns K., Lutgerink J., Van Buuren H. (2014). The role of knowledge sharing self-efficacy in sharing open educational resources. Comput. Hum. Behav..

[bib36] Van Wee B. (2016). Accessible accessibility research challenges. J. Transport Geogr..

[bib37] Venkatesh V., Morris M.G., Davis G.B., Davis F.D. (2003). User acceptance of information technology: toward a unified view. MIS Q..

[bib38] Vermeulen M., Kreijns K., Van Buuren H., Van Acker F. (2017). The role of transformative leadership, ICT-infrastructure and learning climate in teachers' use of digital learning materials during their classes. Br. J. Educ. Technol..

[bib39] Wang J., Tigelaar D.E., Admiraal W. (2019). Connecting rural schools to quality education: rural teachers’ use of digital educational resources. Comput. Hum. Behav..

[bib40] Whitley B.E. (1997). Gender differences in computer-related attitudes and behavior: a meta-analysis. Comput. Hum. Behav..

[bib41] Willaby H.W., Costa D.S., Burns B.D., maccann C., Roberts R.D. (2015). Testing complex models with small sample sizes: a historical overview and empirical demonstration of what partial least squares (PLS) can offer differential psychology. Pers. Indiv. Differ..

[bib42] Yau H.K., Ho T.C. (2015). The influence of subjective norm on Intention to Use in using e-learning: an empirical study in Hong Kong higher education.

[bib43] Yuen A.H.K., Ma W.W.K. (2002). Gender differences in teacher computer acceptance. J. Technol. Teach Educ..

